# Quantum sensors in space: unveiling the invisible universe

**DOI:** 10.1093/nsr/nwaf389

**Published:** 2025-09-22

**Authors:** Yuanhong Wang, Xingming Huang, Min Jiang, Qing Lin, Wenqiang Zheng, Yuan Sun, Liang Liu, Xinhua Peng, Zhengguo Zhao, Jiangfeng Du

**Affiliations:** Laboratory of Spin Magnetic Resonance, Schoo of Physical Sciences, Anhui Province Key Laboratory of Scientific Instrument Development and Application, University of Science and Technology of China, China; Hefei National Laboratory, University of Science and Technology of China, China; Laboratory of Spin Magnetic Resonance, Schoo of Physical Sciences, Anhui Province Key Laboratory of Scientific Instrument Development and Application, University of Science and Technology of China, China; Hefei National Laboratory, University of Science and Technology of China, China; Laboratory of Spin Magnetic Resonance, Schoo of Physical Sciences, Anhui Province Key Laboratory of Scientific Instrument Development and Application, University of Science and Technology of China, China; Hefei National Laboratory, University of Science and Technology of China, China; State Key Laboratory of Particle Detection and Electronics, University of Science and Technology of China, China; Deep Space Exploration Laboratory/Department of Modern Physics, University of Science and Technology of China, China; Deep Space Exploration Laboratory/Department of Modern Physics, University of Science and Technology of China, China; Zhejiang Provincial Key Laboratory and Collaborative Innovation Center for Quantum Precision Measurement, College of Science, Zhejiang University of Technology, China; Zhejiang Provincial Key Laboratory and Collaborative Innovation Center for Quantum Precision Measurement, College of Science, Zhejiang University of Technology, China; Laboratory of Spin Magnetic Resonance, Schoo of Physical Sciences, Anhui Province Key Laboratory of Scientific Instrument Development and Application, University of Science and Technology of China, China; Hefei National Laboratory, University of Science and Technology of China, China; State Key Laboratory of Particle Detection and Electronics, University of Science and Technology of China, China; Deep Space Exploration Laboratory/Department of Modern Physics, University of Science and Technology of China, China; Hefei National Laboratory, University of Science and Technology of China, China

## Abstract

High-speed China Space Station, equipped with in-orbit quantum nuclear-spin sensors, would be a powerful tool for exploring new interactions beyond the Standard Model.

The nature of dark matter and the limitations of the Standard Model have long motivated physicists to explore uncharted territories in particle physics. One of the most promising theoretical candidates for dark matter is ultralight exotic bosons, such as axions [1] and dark photons [2]. Detection approaches for these bosons involve either searches for the dark matter halos they potentially formed in the universe [3] or investigations of exotic interactions they mediated between standard-model fermions [4]. The latter crucially eliminates mass-scanning requirements through broad spectral coverage while avoiding assumptions of model-dependent dark matter abundance [5].

The exotic interactions include 16 terms, 15 of which exhibit spin dependence and 10 demonstrate velocity dependence [5,6], enabling quantum spin sensors to study these interactions from moving polarized spin sources or mass sources. However, detecting these new boson-mediated interactions demands experimental sensitivities far beyond current terrestrial capabilities. Terrestrial search experiments face an insurmountable trade-off: enhancing exotic interaction strength requires increasing the polarized spin number or unpolarized particle number of the source and simultaneously enhancing its relative velocity to the spin sensor, yet these two requirements are always inversely constrained. For instance, spin sources with $10^{25}$ polarized electrons, a benchmark in laboratory experiments, achieve velocities limited to about 20 m/s [7]. This bottleneck has left vast regions of the theoretical parameter space unexplored.

This inspired the Space-based Quantum Sensing for Interaction and Exotic Bosons Research Exploration (SQUIRE) scheme [8]. The ambitious plan deploys quantum spin sensors on space platforms (like the China Space Station [9]) to hunt for pseudomagnetic fields induced by exotic interactions between sensor spins and Earth’s geoelectrons, as seen in Fig. 1. Combining quantum metrology with space technology opens a door to a broad range of investigations in the frontiers of fundamental physics.

**Figure 1.**
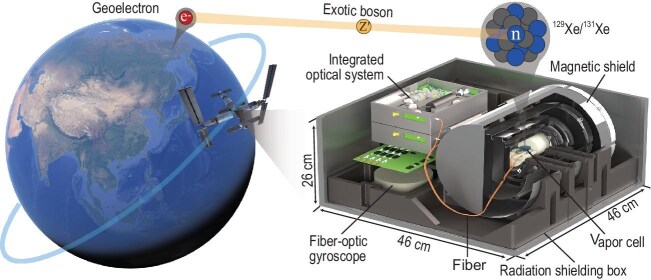
Schematic representation of space-based searches for ultralight exotic bosons and the prototype space quantum sensor.

**The space advantage.** The SQUIRE scheme breaks the bottleneck of inability to boost two key parameters (relative velocity and polarized spin number) for maximizing SSVI searches at the same time. The China Space Station in low Earth orbit, stably orbiting at 7.67 km/s relative to the Earth [9]—close to the first cosmic speed—is nearly 400 times faster than

motion sources in terrestrial experiments [7]. Simultaneously, the Earth itself acts as a massive, naturally polarized spin source [8,10]. Unpaired geoelectrons in the mantle and crust, polarized under the geomagnetic field, yield a staggering $10^{42}$ polarized electron spins—surpassing laboratory $\mathrm{SmCo_{5}}$ spin sources [7] by about $10^{17}$. Moreover, the scheme modulates exotic signals into periodic oscillations through the orbital motion of space platforms. For the China Space Station (with an orbital period of $\sim$1.5 h), the exotic signal is modulated to about 0.189 mHz—a regime where noise is inherently lower.

These characteristics highlight the advantage of space: not only does it amplify the signal, but also modulates it into a detectable form. Simulations predict exotic field amplitudes up to 20 pT for SSVI in the scheme, much stronger than terrestrial limits (0.015 pT) [10] and well within the detection capability of existing spin sensors. Under conservative sensitivity estimation for a space spin sensor of $150\, \text{fT}/\text{Hz}^{1/2}$ and planned detection duration of 100 days, the SQUIRE scheme improves the search sensitivity for all of the SSVI (including $V_{6+7}$, $V_{8}$, $V_{14}$, $V_{15}$ and $V_{16}$) by 6–7 orders of magnitude for the force range ${>}10^6$ m. Such improvement is physically unattainable on Earth; replicating SQUIRE’s sensitivity would require terrestrial spin sources moving at superluminal speeds.

**A prototype space quantum sensor.** Implementing the SQUIRE scheme requires sensors that can withstand the harsh conditions of space while maintaining high sensitivity and long-term stability. Space spin sensors face three primary interference sources: geomagnetic temporal and spatial fluctuations ($\sim\! 20\mu$T), platform-induced vibration ($\sim\! 0.005\, ^{\circ } /\mathrm{s}$) [9], disruptions from cosmic radiation (30 disruptions per day).

To address these challenges, the SQUIRE prototype space sensor incorporates three breakthrough technologies [8].

(i) *Dual noble-gas spin sensor.* By employing isotopes $^{129}$Xe and $^{131}$Xe with opposite gyromagnetic ratios, the sensor suppresses common-mode magnetic noise while maintaining sensitivity to SSVI signals. This technique achieves a $10^4$-fold suppression of magnetic noise, and when combined with the $10^8$-fold suppression provided by multi-layer magnetic shielding, geomagnetic fluctuations can be reduced to below 0.02 fT.

(ii) *Vibration-compensating technique.* The spin sensor is mounted with a fiber-optic gyroscope with a sensitivity of $2\times 10^{-6}\, ^{\circ }/\mathrm{s}$, which suppresses the vibration noise to a negligible 0.65 fT.

(iii)*Radiation-hardened architecture.* A 0.5-cm aluminum enclosure and triple modular redundancy in control circuits mitigate cosmic ray impacts. The latter ensures system functionality even if two of three redundant circuits fail, reducing disruptions to $<$1 per day.

By combining these three technologies, SQUIRE experimentally validates a prototype space sensor that achieves a single-shot sensitivity of 4.3 fT at 1165 s—ideal for detecting SSVI signals with a period of 1.5 h.

**Broader implications.** In addition to exotic interaction searches, the quantum spin sensors installed on the China Space Station enable a broad range of fundamental physics research in space. SQUIRE envisions a ‘space-ground integrated’ network of quantum sensors, linking the orbital sensors with terrestrial counterparts to substantially boost the sensitivity for various dark matter models. Such a network has the potential to significantly enhance sensitivity for various beyond-standard-model physics, including other exotic interactions, axion halo [3] and CPT violation probes. In particular, the high-speed orbital motion would enhance the coupling between axion halo and nucleon spins, achieving a 10-fold improvement in direct dark matter searches compared to terrestrial experiments. Moreover, as plans for deep-space exploration progress, the SQUIRE scheme could stimulate interest in utilizing the resources of distant planets (such as Jupiter and Saturn rich in polarized particles) to explore new frontiers in physics.
